# Diabetic peripheral neuropathy: age-stratified glycemic control

**DOI:** 10.3389/fendo.2024.1377923

**Published:** 2024-04-17

**Authors:** Chi-Sheng Wang, Yen-Wei Pai, Ching-Heng Lin, I-Te Lee, Hsiao-Hui Chen, Ming-Hong Chang

**Affiliations:** ^1^ Neurological Institute, Taichung Veterans General Hospital, Taichung, Taiwan; ^2^ Department of Post-Baccalaureate Medicine and Brain and Neuroscience Research Center, College of Medicine, National Chung Hsing University, Taichung, Taiwan; ^3^ Department of Medical Research, Taichung Veterans General Hospital, Taichung, Taiwan; ^4^ Division of Endocrinology and Metabolism, Department of Internal Medicine, Taichung Veterans General Hospital, Taichung, Taiwan; ^5^ Department of Medicine, School of Medicine, Chung Shan Medical University, Taichung, Taiwan

**Keywords:** age, diabetic peripheral neuropathy, glycemic control, type 2 diabetes mellitus, risk factor

## Abstract

**Background:**

We explore the effect of suboptimal glycemic control on the incidence of diabetic peripheral neuropathy (DPN) in both non-elderly and elderly patients with type 2 diabetes mellitus (T2DM).

**Methods:**

A 6-year follow-up study (2013-2019) enrolled T2DM patients aged >20 without DPN. Participants were classified into two groups: those below 65 years (non-elderly) and those 65 years or older (elderly). Biochemical measurements, including glycated hemoglobin (HbA1C), were recorded regularly. DPN was diagnosed using the Michigan Neuropathy Screening Instrument examination. The outcome was DPN occurrence in 2019.

**Results:**

In 552 enrollments (69% non-elderly), DPN occurred in 8.4% non-elderly and 24.0% elderly patients. A higher initial HbA1C level was significantly linked with a higher risk of future DPN in the non-elderly group (adjusted odds ratio [AOR] 1.46, 95% CI 1.13–1.89, p=0.004). In comparison, HbA1c at the end of the study period was not associated with DPN in the non-elderly group (AOR 1.17, 95% CI 0.72–1.90, p=0.526). In the elderly group, no statistical relationship was found between HbA1C levels and DPN, either in 2013 or in 2019.

**Conclusion:**

Suboptimal glycemic control at baseline, rather than at the end of the study period, predicts an increased risk of future DPN in individuals with T2DM under age 65. This correlation is not seen in elderly patients. Therefore, we recommend implementing enhanced glycemic control early in middle-aged T2DM patients and propose individualized therapeutic strategies for diabetes in different age groups.

## 1 Introduction

Diabetic peripheral neuropathy (DPN), which is one of the most common complication of diabetes mellitus (DM), is an important issue when it comes to DM care. It affects up to 50% of adults with type 2 DM (T2DM) during their lifetime (1). DPN contributes to numerous disabling morbidities, and DPN-related diabetic foot ulcerations are the most common cause of non-traumatic lower-limb amputations in most high-income countries (2, 3). Besides, DPN can also lead to impaired balance, gait disturbance, and distressing neuropathic pain, which result in decrease in quality of life, and are frequently resistant to conventional treatment (4). Therefore, early diagnosis and prevention the development of DPN is crucial.

In real-world practice, the diagnosis of DPN is typically based on patient medical history, clinical examinations, and validated scoring clinical assessments (such as the Michigan Neuropathy Screening Instrument (MNSI), the Toronto Clinical Neuropathy Score, and the United Kingdom Screening Test) (1, 5, 6). MNSI, which consists of assessing the physical appearance of feet, ulceration, ankle deep tendon reflexes, perception of light touch (using the Semmes-Weinstein 5.07 10-g monofilament), and distal vibration (using a 128-Hz tuning fork) (5), is the most widely used diagnostic tool.

Unfortunately, despite the severe complications and significant impact on patients’ daily lives and socio-economic status, DPN often begins insidiously. In the early stage, DPN typically present no discernible symptoms, making it hard to detect until it is well established, at which point it may become challenging to reverse (1). While DPN occurred, there is only disease-modifying therapy that targets the underlying nerve damage: intensive glycemic control in type 1 DM (T1DM) (7, 8), yet it is ineffective type 2 DM (T2DM) (9). The absence of disease-modifying therapy underscores the indispensability of preventive strategy prior to the onset of DPN in diabetes care, particularly in patients with T2DM. Good glycemic control has been demonstrated to reduce the occurrence rate of DPN in patients with T1DM. Interestingly, the same outcome has not been observed in patients with T2DM (7–9). However, in clinical setting, patients who suffer from complications of DM are mainly diagnosed with T2DM. Given that most patients diagnosed with DM complications predominantly have T2DM, there’s considerable research interest in exploring the link between poor glycemic control and subsequent DPN in T2DM. Despite several recent studies on the topic, definitive conclusions remain elusive.

We hypothesized that the definite effect of each predictor of DPN might be diverse in different age groups, especially glycemic control status. Therefore, we aim to investigate the effect of suboptimal glycemic control on the incidence of DPN in Taiwanese adults with T2DM, focusing on both non-elderly and elderly patients.

## 2 Materials and methods

### 2.1 Study design and participants

The study is a hospital-based 6-year follow-up study from a prospective DM registry at a comprehensive medical center located in central Taiwan. The study began in 2013, and its subjects include individuals over 20 years of age who had previously known or were newly diagnosed with type 2 diabetes. Each of the participants was been diagnosed by endocrinologists in the outpatient department of Taichung Veterans General Hospital in adherence to the criteria of American Diabetes Association (ADA). Relevant clinical data was collected at the time of enrollment and during subsequent follow up period, including patient’s medical records, laboratory test results, questionnaires and anthropometric measurements. Exclusion criteria were (1) patients had DPN at baseline; (2) patients having type 1 DM or gestational diabetes and (3) patients with missing data at baseline or at the end of study period.

Participants were monitored through clinical follow-up examinations and questionnaires. Our study was conducted until 2019, 6 years after its commencement. The primary outcome was the occurrence of DPN in 2019. Patients were classified into two age groups in 2013: (1) the elderly group, consisting of patients aged 65 years and above, and (2) the non-elderly group, comprising patients under 65 years old. We further defined the patients who developed DPN during follow-up period as the “incident DPN” sub-group. The sociodemographic factors and biochemical factors at baseline (2013) and at the end of study period (2019) between the patients with and without incident DPN were compared in each age group. The study mainly concentrated on the glycemic control status.

Patient’s information was anonymized through a computer system, and researchers were blinded to this data. Our study received approval from the Institutional Review Board at Taichung Veterans General Hospital (CG18082B-1). All participants volunteered for the current studies, and provided written informed consent prior to enrolment.

### 2.2 Assessment of diabetic peripheral neuropathy, biochemical data and glycemic control

All study participants underwent a DPN assessment at enrollment and a second assessment after six years of follow-up by the same trained and certified nursing staff. DPN was evaluated based on MNSI examination (MNSIE), the second component of MNSI. In line with previous validated studies on adults, individuals whose MNSIE score > 2 were diagnosed with DPN (5).

Laboratory tests were conducted during outpatient follow-ups. Blood samples were collected in the morning after an overnight fasting period from the antecubital vein. Fasting plasma glucose (FPG; using standard enzymatic methods), glycated hemoglobin (HbA1c; using high-performance liquid chromatography), serum creatinine concentration and plasma lipid profiles (using standard enzymatic methods), including total cholesterol (TC), high-density lipoprotein (HDL), low-density lipoprotein (LDL), and triglyceride (TG). The blood test was performed at least once every three months.

We defined glycemic control status at baseline using the mean HbA1c level in 2013 and glycemic control status at the end of the study period with the mean HbA1c in 2019. All parameters were measured at least four times both in 2013 and 2019. After which, their respective averages were calculated.

### 2.3 Anthropometric measurements and statistical methods

All participants received anthropometric measurements, including height, weight, waist circumference, and blood pressure by our collaborated case-management nurse in 2013 and 2019, respectively. Smoking status and medication usage were also recorded. Current medication usage including oral hypoglycemic agent (OHA), insulin, antihypertensive drugs and lipid-lowering drugs such as statins and fibrates. The number of insulin used is defined as the number of individuals who use insulin divided by the total number of individuals within each age group, expressed as the median (interquartile range [IQR]). Comorbidities obtained from medical record and based on International Classification of Diseases, 9^th^ revision Clinical Modification (ICD-9-CM) and 10^th^ revision (ICD-10) which including hypertension (ICD-9-CM codes 401-405, ICD-10 codes I10-I15), cerebrovascular disease (ICD-9-CM codes 430-438, ICD-10 codes I60-I69), ischemic heart disease (ICD-9-CM codes 410-414, ICD-10 codes I20-I25), liver disease (ICD-9-CM codes 571-573, ICD-10 codes K70-K77). For the details of anthropometric measurements, please refers to our published study (10, 11).

### 2.4 Statistical methods

Continuous data were presented as mean values ± standard deviation (SD) and categorical data as numbers with percentages, while discrete variables were expressed as median and IQR. We used Fisher’s exact test or the Chi-square test to analyze categorical variables. While discrete variable analyses were performed using the Mann-Whitney U test and continuous variable analyses were performed using the independent t-test. *p* values less than 0.05 were considered significant. The multivariable logistic regression analysis was performed in each age group to investigate the impact of each independently identified variable on the incident DPN. The multivariable regression model includes all confounders and the adjusted odds ratios (AOR) with 95% confidence interval (CI) were calculated between the comparison groups.

We conduct two multivariable regression models for each age group: applied parameters in 2013 and parameters in 2019, respectively. These two models adjusted all the confounders, besides, HbA1C would be included even if descriptive statistics revealed no statistical significant.

Because the relationship between HbA1c and FPG is hyperbolic, we do not adjust one for each other to statistic the interference between baseline glycemic control and the occurrence of DPN.

## 3 Results

**Figure 1:**
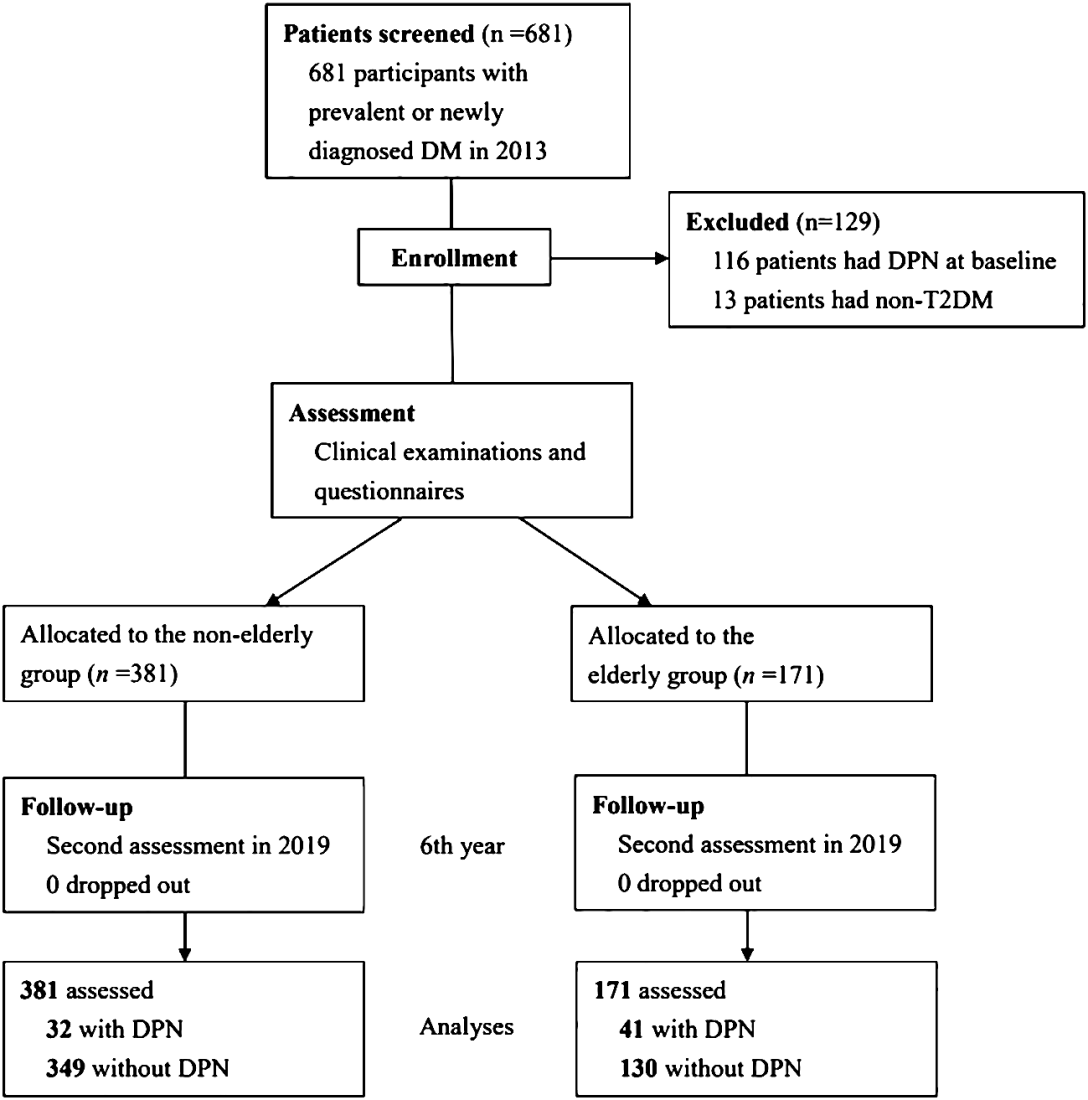
Study flow diagram.

In 2013, we recruited 681 participants with DM as a baseline. Of those participants, 116 (17.0%) with DPN in their initial state and 13 (1.9%) with non-T2DM were excluded. Accordingly, 552 patients were considered eligible to participate in the study (**Figure 1**). That cohort includes 381 patients in the non-elderly group and 171 patients in the elderly group. In total cohort, the participants’ median age was 59.7 ± 10.7 years; the mean duration of DM was 15.2 ± 6.9 years, and the participants’ mean HbA1c level was 7.4 ± 1.3%. Seventy-three patients developed DPN during follow-up period, and the cumulative incidence of DPN over the 6 years of follow-up was 13.2%.

### 3.1 Characteristics of patients with and without incident DPN in each age group at baseline

We compared the sociodemographic and biochemical factors at baseline for patients with and without incident DPN in both age groups (**Table 1**). In addition, the baseline sociodemographic and biochemical factors of our cohort were shown in **Supplementary Table 1**.

**Table 1:** Characteristics of patients with incident DPN and without incident DPN in non-elderly and elderly groups at baseline (2013).

Variable	Non-elderly group, age <65 at baseline (n=381)		p value	Elderly group, age ≥ 65 at baseline (n=171)		p value
	Without incident DPN (n=349)	Incident DPN (n=32)		Without incident DPN (n=130)	Incident DPN (n=41)	
Sociodemographic factors						
Age, years, mean(SD)	54.2(7.6)	56.0(6.4)	0.196	71.3(5.6)	73.0(6.6)	0.109
Male gender, n(%)	210(60.2)	27(84.4)	0.007	61(46.9)	32(78.1)	<0.001
Height, cm, mean(SD)	164.1(8.2)	166.7(8.3)	0.090	159.9(7.6)	164.6(7.8)	0.001
Weight, kg, mean(SD)	70.1(13.4)	75.3(12.5)	0.038	63.4(11.3)	69.9(9.7)	0.001
Waist circumference, cm, mean(SD)	89.7(11.0)	87.7(9.5)	0.637	88.9(9.3)	92.9(7.9)	0.208
SBP, mmHg, mean(SD)	129.4(12.8)	133.6(12.5)	0.073	132.1(13.8)	133.4(12.6)	0.587
DBP, mmHg, mean(SD)	78.2(8.9)	80.6(8.6)	0.141	75.7(11.0)	74.6(7.3)	0.482
Smoker, n(%)	45(12.9)	8(25.0)	0.066	9(6.9)	2(4.9)	1.000
Duration of diabetes, years, mean(SD)	13.7(6.1)	16.8(7.7)	0.009	18.2(7.7)	17.9(6.3)	0.833
Number of OHA used, mean(SD)	1.8(1.0)	1.8(1.1)	0.927	2.1(1.0)	2.9(4.6)	0.285
Number of insulin used, mean(IQR)	0.3(0-0.5)	0.6(0-0.9)	0.008	0.2(0-0.5)	0.1(0-0.3)	0.720
Hypertension, n(%)	206(59.0)	20(62.5)	0.702	105(80.8)	35(85.4)	0.505
Cerebrovascular disease, n(%)	51(14.6)	6(18.8)	0.603	30(23.1)	13(31.7)	0.267
Ischemic heart disease, n(%)	51(14.6)	4(12.5)	1.000	18(13.9)	7(17.1)	0.610
Liver disease, n(%)	50(14.3)	6(18.8)	0.444	16(12.3)	5(12.2)	0.985
Biochemical factors						
FPG, mg/dL, mean(SD)	140.5(38.7)	163.3(65.6)	0.071	139.8(36.2)	131.7(28.4)	0.195
HbA1c, %, mean(SD)	7.4(1.3)	8.6(2.0)	0.002	7.2(1.0)	6.9(0.8)	0.093
UACR, mg/g, mean(SD)	68.0(247.7)	128.5(225.5)	0.240	85.1(287.6)	70.9(172.9)	0.783
TG, mg/dL, mean(SD)	143.5(138.8)	227.2(299.6)	0.155	118.3(65.8)	126.1(76.0)	0.553
HDL-C, mg/dL, mean(SD)	51.8(15.0)	48.4(15.7)	0.250	54.4(14.7)	48.4(18.2)	0.051
LDL-C, mg/dL, mean(SD)	101.3(31.6)	111.7(48.9)	0.298	96.1(23.7)	84.6(26.4)	0.014
TC, mg/dL, mean(SD)	168.2(34.3)	181.5(53.7)	0.211	163.3(27.8)	149.8(32.8)	0.018
Creatinine, mg/dL, mean(SD)	0.9(0.3)	1.1(0.4)	0.020	1.02(0.4)	1.08(0.3)	0.414
GPT, U/L, mean(SD)	35.9(27.1)	40.0(22.9)	0.455	26.8(15.6)	30.7(36.6)	0.528

Among non-elderly group, male gender (84.4% vs. 60.2%, p=0.007), body weight (75.3 ± 12.5 kg vs. 70.1 ± 13.4 kg, p< 0.05), HbA1C (8.6 ± 2.0% vs. 7.4 ± 1.3%, p=0.002), duration of diabetes (16.8 ± 7.7 years vs. 13.7 ± 6.1 years, p=0.009), number of insulin used (0.8 vs. 0.6, p=0.008) and serum creatinine (1.1 ± 0.4 mg/dL vs. 0.9 ± 0.3 mg/dL, p= 0.02) were significantly higher at baseline in patients with incident DPN than in those without incident DPN.

Among elderly group, male gender (78.1% vs. 46.9%, p<0.001), height (164.6 ± 7.8 cm vs. 159.9 ± 7.6 cm, p= 0.001) and body weight (69.9 ± 9.7 kg vs. 63.4 ± 11.3 kg, p= 0.001) were significantly higher at baseline in patients with incident DPN than in those without incident DPN. LDL (84.6 ± 26.4 mg/dL vs. 96.1 ± 23.7 mg/dL, p=0.014) and TC (84.6 ± 26.4 mg/dL vs. 163.3 ± 27.8 mg/dL, p=0.018) were significantly lower at baseline in patients with incident DPN than in those without incident DPN.

### 3.2 Characteristics of patients with and without incident DPN in each age group in 2019

**Table 2** summarizes the sociodemographic and biochemical factors of the patients with and without incident DPN in both age groups in 2019.

**Table 2:** Characteristics of patients with incident DPN and without incident DPN in non-elderly and elderly groups at the end of study period (2019).

Variable	Non-elderly group, age <65 at baseline (n=381)		p value	Elderly group, age ≥ 65 at baseline (n=171)		p value
	Without DPN (n=349)	With DPN (n=32)		Without DPN (n=130)	With DPN (n=41)	
Sociodemographic factors						
Age, years, mean(SD)	54.2(7.6)	56.0(6.4)	0.196	71.3(5.6)	73.0(6.6)	0.109
Male gender, n(%)	210(60.2)	27(84.4)	0.007	61(46.9)	32(78.1)	<0.001
Height, cm, mean(SD)	163.8(8.4)	166.2(8.3)	0.110	158.2(8.0)	162.6(8.7)	0.003
Weight, kg, mean(SD)	69.3(14.1)	75.9(12.7)	0.011	61.4(11.4)	69.5(10.1)	<0.001
Waist circumference, cm, mean(SD)	89.7(11.0)	93.9(9.5)	0.038	87.5(9.6)	94.6(9.4)	<0.001
SBP, mmHg, mean(SD)	132.3(16.8)	134.7(17.0)	0.451	136.2(16.7)	138.1(15.5)	0.518
DBP, mmHg, mean(SD)	75.8(11.0)	77.2(10.5)	0.510	67.7(11.0)	68.2(9.7)	0.805
Smoker, n(%)	45(12.9)	8(25.0)	0.066	9(6.9)	2(4.9)	1.000
Duration of diabetes, years, mean(SD)	19.7(6.1)	22.8(7.7)	0.009	24.2(7.7)	23.9(6.3)	0.833
Number of OHA used, mean(SD)	1.8(1.0)	1.8(1.1)	0.927	2.1(1.0)	2.9(4.6)	0.285
Number of insulin used, mean(IQR)	0.3(0-0.6)	0.6(0.2-0.9)	0.008	0.2(0-0.5)	0.1(0-0.4)	0.720
Hypertension, n(%)	206(59.0)	20(62.5)	0.702	105(80.8)	35(85.4)	0.505
Cerebrovascular disease, n(%)	51(14.6)	6(18.8)	0.603	30(23.1)	13(31.7)	0.267
Ischemic heart disease, n(%)	51(14.6)	4(12.5)	1.000	18(13.9)	7(17.1)	0.610
Liver disease, n(%)	50(14.3)	6(18.8)	0.444	16(12.3)	5(12.2)	0.985
Biochemical factors						
FPG, mg/dL, mean(SD)	139.9(22.2)	145(21.2)	0.214	141.5(20.0)	141.5(19.6)	0.994
HbA1c, %, mean(SD)	7.4(0.9)	7.8(0.8)	0.016	7.3(0.7)	7.2(0.7)	0.836
UACR, mg/g, mean(SD)	120(476.0)	445.7(1003.6)	0.079	288.9(781.7)	119.2(215.4)	0.268
TG, mg/dL, mean(SD)	118.4(74.3)	195.0(310.9)	0.174	112.1(70.3)	107.3(47.5)	0.622
HDL-C, mg/dL, mean(SD)	51.9(13.8)	48.3(20.9)	0.337	53.1(14.4)	48.7(14.2)	0.086
LDL-C, mg/dL, mean(SD)	84.3(24.3)	78.1(25.7)	0.171	80.6(21.2)	83.2(26.6)	0.581
TC, mg/dL, mean(SD)	152.4(30.5)	157.1(54.8)	0.639	151.8(27.7)	146.4(30.0)	0.288
Creatinine, mg/dL, mean(SD)	1.0(0.8)	1.5(1.2)	0.041	1.3(0.8)	1.2(0.4)	0.362
GPT, U/L, mean(SD)	25.3(15.9)	24.5(11.8)	0.774	20.3(11.6)	21.2(10.2)	0.657

As of 2019, among non-elderly group, male gender (84.4% vs. 60.2%, p=0.007), body weight (75.9 ± 12.7 kg vs. 69.3 ± 14.1 kg, p=0.011), waist circumference (93.9 ± 9.5 cm vs. 89.7 ± 11.0 cm, p=0.038), HbA1C (7.8 ± 0.8% vs. 7.4 ± 0.9%, p=0.016), duration of diabetes (22.8 ± 7.7 years vs. 19.7 ± 6.1 years, p=0.009), number of insulin used (0.8 vs. 0.6, p=0.008) and serum creatinine (1.5 ± 1.2 mg/dL vs. 1.0 ± 0.8 mg/dL, p= 0.041) were significantly higher in patients with incident DPN than in those without incident DPN.

Among elderly group, male gender (78.1% vs. 46.9%, p<0.001), height (162.8 ± 8.7 cm vs. 158.2 ± 8.0 cm, p=0.003), body weight (69.5 ± 10.1 kg vs. 61.4 ± 11.4 kg, p<0.001) and waist circumference (94.6 ± 9.4 cm vs. 87.5 ± 9.6 cm, p<0.001) were significantly higher in patients with incident DPN.

### 3.3 Multivariable logistic regression model

In **Tables 3**, **4**, we present results from our multivariable logistic regression model which respectively applied the confounders and HbA1C levels from baseline and 2019; the data are presented as AOR for the risk factors of incident DPN.

**Table 3:** Risk factors of future DPN in multivariable logistic regression at baseline.

Table 3-1. Non-elderly group, age <65			
Variables	Adjusted Odds ratio^a^	95% CI	p value
Male	2.00	(0.58-6.89)	0.272
Weight, kg	1.03	(0.99-1.07)	0.074
HbA1c, % (mmol/mol)	1.46	(1.13-1.89)	0.004
Duration of diabetes, years	1.07	(1.00-1.14)	0.041
Number of insulin used	0.83	(0.42-1.61)	0.571
Creatinine, mg/dL	4.41	(1.05-18.51)	0.042
Table 3-2. Elderly group, age ≥65			
Male	2.32	(0.58-9.33)	0.234
Height, cm	1.01	(0.93-1.11)	0.761
Weight, kg	1.05	(0.99-1.10)	0.062
LDL-C, mg/dL	0.97	(0.95-0.99)	0.025
TC, mg/dL	1.01	(0.99-1.03)	0.592
HbA1c, % (mmol/mol)	0.87	(0.53-1.42)	0.570

a Multivariable logistic regression was adjusted for all variables in **Tables 3-1, 3-2**, respectively.

**Table 4:** Risk factors of DPN in multivariable logistic regression at the end of study period (2019).

Table 4-1. Non-elderly group, age <65			
Variables	Adjusted Odds ratio^a^	95% CI	p value
Male	3.24	(0.99-10.59)	0.052
Weight, kg	1.03	(0.97-1.10)	0.352
Waist circumference, cm	0.99	(0.92-1.08)	0.896
HbA1c, % (mmol/mol)	1.17	(0.72-1.90)	0.526
Duration of diabetes, years	1.06	(1.00-1.13)	0.051
Number of insulin used	1.27	(0.69-2.34)	0.442
Creatinine, mg/dL	1.18	(0.87-1.59)	0.287
Table 4-2. Elderly group, age ≥65			
Male	2.46	(0.78-7.75)	0.124
Height, cm	1.02	(0.94-1.10)	0.688
Weight, kg	0.99	(0.92-1.07)	0.867
Waist circumference, cm	1.07	(0.99-1.16)	0.071
HbA1c, % (mmol/mol)	0.99	(0.57-1.71)	0.964

a Multivariable logistic regression was adjusted for all variables in **Tables 4-1, 4-2**, respectively.

In the non-elderly group, increased HbA1C level at baseline (AOR 1.46, 95% CI 1.13–1.89, p=0.004) was significantly associated with an increased risk of DPN. The duration of DM (AOR 1.07, 95% CI 1.00–1.14, p=0.041) and serum creatinine level (AOR 4.41, 95% CI 1.05–18.51, p=0.042) were significantly associated with a higher risk of DPN as well. On the other hand, male gender, body weight, mean number of insulin used at baseline and all parameters in 2019 revealed no statistically significant associations with the risk of DPN after adjustment for all confounding factors (**Tables 3-1**, **4-1**).

In the elderly group, lower LDL at baseline (AOR 0.97, 95% CI 0.95–0.99, p=0.025) was significantly associated with an increased risk of DPN. On the other hand, HbA1C level, male gender, height, weight, TC at baseline and all parameters in 2019 revealed no statistically significant associations with the risk of DPN (**Tables 3-2**, **4-2**).

## 4 Discussion

To the best of our knowledge, our study is the first large-scale follow-up study to investigate risk factors for DPN across different age groups. The main findings of our study can be summarized as follows: (1) suboptimal glycemic control status at baseline is a predictor of future DPN in Taiwanese individuals with T2DM under the age of 65 but not in elderly populations, (2) glycemic control status at the end of study period did not significantly correlate with DPN risk in either age group, (3) the predictors of future DPN may differ among varying age populations.

In our study, the cumulative incidence of DPN was 8.4% and 24.0% in the non-elderly and elderly groups, respectively. Those data were based on 6 years of follow-up using the MNSIE for DPN diagnosis. The incidence of DPN in the present study is comparable to previous large-scale studies conducted in Han Chinese populations (n = 37,375, annual incidence of 3.2%) (12) but lower than that reported in Western populations (13, 14). The discrepancy might be attributed to the ethnic makeup of our study group and their relatively better control of blood sugar levels.

### 4.1 The association between glycemic control and incident DPN

It is a topic of ongoing research whether intensive glycemic control can prevent patients with T2DM from developing DPN. Previous randomized controlled trials have demonstrated intensive glycemic control can substantially reduce the incidence of DPN in T1DM populations (7, 8). Furthermore, long-term poor glycemic control in T1DM patients is known to be a determining factor in rapid DPN development (15). In contrast, intensive glycemic control has revealed limited effectiveness in preventing DPN in T2DM patients (9). This observation remains true, regardless of whether the DPN was diagnosed using the MNSI (16) or nerve conduction study (17). Correspondingly, previous large-scale studies support the concept that baseline HbA1c levels are not an independent risk factor for DPN across all age groups (10, 18).

However, in clinical practice, T2DM is the most prevalent form of DM, and more than 90% of DPN cases can be attributed to T2DM (19). Therefore, it garners significant attention that whether suboptimal glycemic control is a predictor of future DPN in individuals with T2DM. Our study confirms this correlation in patients under the age of 65.

To date, the pathogenesis of neurotoxicity in patients with diabetes who have poor glycemic control is well-established. Hyperglycemia, by disrupting various metabolic pathways, gives rise to irregularities within nerve-related processes that specifically involve the polyol, hexosamine, and protein kinase C pathways (20). These irregularities serve as triggers for the release of proinflammatory cytokines, the accumulation of advanced glycation end products, and the generation of reactive oxygen species. Meanwhile, microangiopathic alterations in the vasa nervorum can lead to the condition of neuroischemia (21). This sequence of events results in oxidative stress in the nervous system and neuron apoptosis, which contribute to the development of DPN.

We posit that the pathogenesis of neurotoxicity generated by poor glycemic control should not differ depending on whether a patient has T1DM or T2DM. Therefore, we propose the hypothesis that suboptimal glycemic control contributing to DPN may vary depending on the individual with diabetes. Concurrently, our previous research findings have indicated that DPN in elderly patients may be attributed to multitude of factors, including advanced age itself and the relatively long duration of DM (10). In present study, we accordingly stratified our subjects at the age of 65 to assess the impact of glycemic control on the incidence of DPN among non-elderly and elderly patients.

Furthermore, our finding might be affected by the known link between age and HbA1c levels. One potential explanation is that older individuals physiologically have lower red blood cell counts, rendering HbA1c an inadequate indicator for evaluating glycemic control in a geriatric population (22, 23). When it comes to the glycemic control status at the end of the study period, there was no significant association between the 2019 HbA1c levels and DPN risk in either the non-elderly or elderly groups. This finding seems to imply that, as diabetes progresses over time, enhanced glycemic control becomes insufficient in preventing DPN. Our findings underscore the importance of early prompt intervention. Implementing enhanced glycemic control as a preventive strategy against DPN should commence as early as possible, particularly in non-elderly individuals.

### 4.2 Other risk factors of future DPN between age groups

Upon adjusting for potential confounding factors, our data revealed elevated risk of DPN was linked to the duration of DM and serum creatinine levels at baseline in the non-elderly group. In the elderly group, a lower baseline LDL was linked to an increased DPN risk. Our findings are consistent with previous literature and our published study (11, 18, 24, 25).

Regarding dyslipidemia, we also found that lower LDL at baseline was associated with a risk of DPN in the elderly group. Previous clinical studies have demonstrated inconsistent results in terms of a correlation between DPN and serum lipid profiles (26, 27). Nevertheless, the association between dyslipidemia and neurotoxicity is predominantly documented in preclinical research (28, 29). One possible explanation for the association between lower LDL levels and increased risk of incident DPN in the elderly group may stem from the fact that, in older adults, lower LDL levels might reflect underlying frailty, chronic illness, chronic inflammation, or malnutrition, thereby increasing their vulnerability to neuropathic complications (30, 31). Our study primarily investigates that whether glycemic control status is associated with incident DPN among different age groups. Further research on dyslipidemia is warranted to elucidate the underlying mechanisms and determine the clinical implications for DPN preventive strategies.

In addition, HbA1c and serum creatinine levels are crucial in clinical practice because of the “modifiable” characteristic and they are “commonly” available assessment. These properties make them suitable targets for therapeutic intervention. Therefore, we recommend early management of poor glycemic control and impaired renal function should be integral to diabetes-treatment strategies in middle-aged patients to prevent DPN. In addition, HbA1c and serum creatinine levels can serve as effective monitoring indicators.

The strengths of our study include the innovative age-stratified design, the large-scaled and unselected nature of the participants, the long-term follow-up period, and the inclusion of several potential risk factors at baseline. Nevertheless, our study has several limitations. First, we did not adhere to an interval-based examination protocol to assess the occurrence of DPN. We accordingly could not perform time-to-event analysis. Second, the inclusion of participants from a single hospital potentially restricted the generalizability of our findings. Third, the number of participants might be insufficient for powered subgroup analyses (for example, only 171 patients in elderly group). Fourth, we abstained from employing confirmatory diagnostic measures such as nerve conduction studies for detecting DPN. It is noteworthy, however, per ADA guidelines, DPN diagnosis usually relies on clinical evaluation – specifically, the highly sensitive and specific MNSI clinical screening tool. Lastly, our study did not include factors like alcohol consumption, heavy metal exposure, or non-hypoglycemic drugs, which are known confounding factors for neuropathy, thus representing a limitation. Despite these issues, similar prevalence rates of comorbidities between subgroups with and without incident DPN across both age groups may suggest no significant differences in routine medication usage among participants.

## 5 Conclusion

Suboptimal glycemic control at baseline is a predictor of future DPN in adult patients with T2DM who are less than 65 years old but not in elderly populations. Other risk factors include the duration of DM and serum creatinine levels at baseline in patients under the age of 65, as well as low LDL levels at baseline in elderly patients. Intensive glycemic control should be provided to middle-aged patients as soon as possible to prevent the occurrence of future DPN and HbA1c levels can serve as an appropriate and effective monitoring indicators. Furthermore, we recommend tailoring treatment strategies to the individual patient’s age group. Our findings provide valuable insights for researchers studying the pathogenesis of DPN, and we are hopeful they will encourage the development of disease-modifying treatments in the future.

## Data availability statement

The original contributions presented in the study are included in the article/**Supplementary Material**. Further inquiries can be directed to the corresponding authors.

## Author contributions

C-SW: Conceptualization, Data curation, Formal Analysis, Methodology, Project administration, Resources, Writing – original draft, Writing – review & editing. Y-WP: Conceptualization, Investigation, Methodology, Project administration, Resources, Validation, Writing – review & editing. C-HL: Data curation, Formal Analysis, Resources, Writing – original draft, Visualization. I-TL: Data curation, Resources, Writing – review & editing. H-HC: Formal Analysis, Software, Writing – original draft. M-HC: Conceptualization, Funding acquisition, Investigation, Methodology, Project administration, Resources, Supervision, Validation, Visualization, Writing – review & editing.
