# Supplementary material for: Diabetic peripheral neuropathy: age-stratified glycemic control
Source: Front Endocrinol (Lausanne). 2024 Apr 17;15:1377923. doi: 10.3389/fendo.2024.1377923 (PMC11061506; doi:10.3389/fendo.2024.1377923)
Supplement: Supplementary file 1 [file Table_1.docx]

Supplementary Material

Diabetic Peripheral Neuropathy: Age-stratified Glycemic Control

Chi-Sheng Wang, Yen-Wei Pai^*^, Ching-Heng Lin, I-Te Lee, Hsiao-Hui Chen, Ming-Hong Chang*

*** Correspondence:**

Ming-Hong Chang

Neurological Institute, Taichung Veterans General Hospital, No. 1650, Taiwan Boulevard, Sec. 4, Taichung City 40705, Taiwan

Email: [cmh500809@gmail.com](mailto:cmh500809@gmail.com)

Tel: +886-4-23592525 ext. 3021

***Co-correspondence author**

Yen-Wei Pai

Neurological Institute, Taichung Veterans General Hospital, No. 1650, Taiwan Boulevard, Sec. 4, Taichung City 40705, Taiwan

E-mail: jasminezxcv@gmail.com

Tel: +886-4-23592525 ext. 3025

# Supplementary Figures and Tables

Supplementary Table 1. The sociodemographic and biochemical factors of total cohort at baseline (2013)

| Variable | Total (n=552) | Without incident DPN (n=479) | With incident DPN  (n=73) | *P* value |
| --- | --- | --- | --- | --- |
| **Sociodemographic factors** | | | | |
| Age, years, mean(SD) | 59.7(10.7) | 58.8(10.4) | 65.5(10.7) | <0.001 |
| Male gender, n(%) | 332(60.1) | 272(56.8) | 60(82.2) | <0.001 |
| Height, cm, mean(SD) | 163.3(8.3) | 163(8.3) | 165.6(8.1) | 0.014 |
| Weight, kg, mean(SD) | 68.9(13) | 68.3(13.2) | 72.3(11.3) | 0.016 |
| Waist circumference, cm, mean(SD) | 89.6(10.2) | 89.5(10.4) | 90.8(8.7) | 0.630 |
| SBP, mmHg, mean(SD) | 130.6(13.1) | 130.1(13.1) | 133.5(12.5) | 0.039 |
| DBP, mmHg, mean(SD) | 77.8(8.2) | 77.9(8.1) | 77.3(8.4) | 0.558 |
| Smoker, n(%) | 64(11.6) | 54(11.3) | 10(13.7) | 0.547 |
| Duration of diabetes, years, mean(SD) | 15.2(6.9) | 14.9(6.9) | 17.4(6.9) | 0.004 |
| Number of OHA used, mean(SD) | 1.9(1.0) | 1.9(1.0) | 2.1(1.1) | 0.245 |
| Number of insulin used, mean(IQR) | 0.3(0-0.6) | 0.2(0-0.5) | 0.3(0-0.7) | 0.218 |
| Hypertension, n(%) | 366(66.3) | 311(64.9) | 55(75.3) | 0.080 |
| Cerebrovascular disease, n(%) | 100(18.1) | 81(16.9) | 19(26.0) | 0.060 |
| Ischemic heart disease, n(%) | 80(14.5) | 69(14.4) | 11(15.1) | 0.881 |
| Liver disease, n(%) | 77(13.9) | 66(13.8) | 11(15.1) | 0.767 |
| **Biochemical factors** | | | | |
| FPG, mg/dL, mean(SD) | 141(39.8) | 140.3(38.0) | 145.2(50.1) | 0.439 |
| HbA1c, %, mean(SD) | 7.4(1.3) | 7.3(1.2) | 7.6(1.7) | 0.131 |
| UACR, mg/g, mean(SD) | 75.7(251.7) | 72.8(259.1) | 94.9(196.8) | 0.440 |
| TG, mg/dL, mean(SD) | 141.1(138.5) | 136.7(123.7) | 169.6(209) | 0.220 |
| HDL-C, mg/dL, mean(SD) | 52(15.3) | 52.5(14.9) | 48.4(16.9) | 0.048 |
| LDL-C, mg/dL, mean(SD) | 99.3(31.1) | 99.8(29.7) | 95.8(39.4) | 0.433 |
| TC, mg/dL, mean(SD) | 166.5(34.6) | 166.9(32.7) | 164.1(45.9) | 0.647 |
| Creatinine, mg/dL, mean(SD) | 0.9(0.3) | 0.9(0.3) | 1.1(0.4) | 0.001 |
| GPT, U/L, mean(SD) | 33.5(25.9) | 33.4(24.8) | 34.4(32.0) | 0.807 |

Abbreviations: DPN, diabetic peripheral neuropathy; SD, standard deviation; SBP, systolic blood pressure; DBP, diastolic blood pressure; OHA, oral hypoglycemic agent; IQR, interquartile range; FPG, Fasting plasma glucose; HbA1c, glycated hemoglobin; UACR, urine albumin-creatinine ratio; TG, triglyceride; HDL-C, high-density lipoprotein cholesterol; LDL-C, low-density lipoprotein cholesterol; TC, total cholesterol; GPT, alanine aminotransferase
